# Supplementary material for: Chromothripsis during telomere crisis is independent of NHEJ, and consistent with a replicative origin
Source: Genome Res. 2019 May;29(5):737–49. doi: 10.1101/gr.240705.118 (PMC6499312; doi:10.1101/gr.240705.118)
Supplement: Supplemental Material [file supp_gr.240705.118_Supplemental_file_1.zip › contigs/annotated_contigs/DB51/contig.2.DB51_length_382_mean_cov_4.31937172775.docx]

**DB51_length_382_mean_cov_4.31937172775**

```
TGAGAATCTAATGCCTGAACATCTGTCTCACCCCCAGATGGGACTGGCTAGTTGTAGGAAAACAAACTCAGGGCTCCCACTGATTCTAC
 >chr18:52573188-52573405 + E=3e-113
ATTATAGTGAGTTTATAATTATTTTATTATACATTACAATGTAATAAAAATAGAAATAAATGTAATGCACTTGAATCACACCGAAACCA

TCCTCTCCACCCTGGTCCATGGAAAAACTGTCTT|CCATG|CCAGGCTAATTTCCCAGGACAATGTGGAGTAATCCAAGTGGATGACTC
                                   >chr6:65413478-65413648 + E=7e-85
TTTGGTTCATTACAACATTCATGAAGAGGGAATTTTCAGTGTCAAGGTTCATTGAACATTGATGCATTTAAATATATAATCTCACCACG

CTAACTGTGGTAATTGTTATTACAAGAA
```
